# Prognostic impact of glim-defined malnutrition and phase angle in patients with digestive tumors

**DOI:** 10.3389/fnut.2026.1836804

**Published:** 2026-05-13

**Authors:** María González-Pacheco, Carlos López-Pereira, Arnau Gramage-Viñets, Almudena Lara-Barea, Rocío Fernández-Jiménez, Francisco Javier Vílchez-López

**Affiliations:** 1 Department of Endocrinology and Nutrition, Puerta del Mar University Hospital, Cádiz, Spain; 2 Instituto de Investigación e Innovación Biomédica de Cádiz (INiBICA), Hospital Universitario Puerta del Mar, Universidad de Cádiz, Cádiz, Spain; 3 PhD Program in Clinical Medicine and Public Health, University of Granada, Granada, Spain; 4 Department of Endocrinology and Nutrition, Virgen de la Victoria University Hospital, Málaga, Spain; 5 IBIMA Plataforma BIONAND, Instituto de Investigación Biomédica de Málaga, Spain; 6 Department of Endocrinology and Nutrition, Quironsalud Málaga Hospital, Málaga, Spain; 7 Department of Medicine and Dermatology, Faculty of Medicine, University of Málaga, Málaga, Spain

**Keywords:** digestive cancer, GLIM criteria, malnutrition, morphofunctional assessment, mortality, phase angle

## Abstract

**Background/Objectives:**

Disease-related malnutrition is frequent among patients with digestive neoplasms and is associated with poorer prognosis. The Global Leadership Initiative on Malnutrition (GLIM) criteria have been proposed to standardize its diagnosis; however, their prognostic value in combination with morphofunctional parameters remains insufficiently defined. This study aimed to analyze the association between GLIM-defined malnutrition, phase angle (PhA), and overall survival in patients with digestive cancer.

**Methods:**

We conducted a retrospective longitudinal study including 131 adult patients with digestive neoplasms. Nutritional status was established according to GLIM criteria. Morphofunctional assessment consisted of bioelectrical impedance analysis with PhA determination, muscle ultrasound, and handgrip strength. Overall survival was evaluated using Kaplan–Meier curves and Cox regression models adjusted for age and sex.

**Results:**

Significant differences between the three GLIM categories were observed for several bioimpedance-derived parameters, including body cell mass (BCM), fat-free mass (FFM), and appendicular skeletal muscle mass (ASMM) (*p <* 0.001). PhA was independently associated with GLIM-defined nutritional severity (OR = 2.51; 95% CI: 1.26–4.99; *p* = 0.009), and a cutoff of 3.8° discriminated survival (*p <* 0.05). In the multivariable model, malnutrition was independently associated with higher mortality (HR = 4.35; 95% CI: 1.04–16.67; *p* = 0.044), while PhA showed a protective association (HR = 0.57; 95% CI: 0.34–0.97; *p* = 0.039).

**Conclusion:**

GLIM-defined malnutrition is independently associated with overall survival in patients with digestive cancer. Phase angle provides additional prognostic information and improves risk stratification.

## 1 Introduction

Digestive neoplasms, including colorectal, gastric, pancreatic, esophageal, and biliary tract cancers, remain among the leading causes of cancer morbidity and mortality worldwide (1, 2). Despite advances in cancer treatments and supportive care, overall survival remains limited, especially in advanced stages. Although prognosis has traditionally been based on tumor factors such as stage, histology, and response to treatment, increasing evidence suggests that host-related factors, particularly nutritional status, significantly influence clinical outcomes and survival (3).

Disease-related malnutrition is highly prevalent in digestive neoplasms as a result of direct involvement of the gastrointestinal tract, the presence of symptoms with nutritional impact, systemic inflammation, and toxicity associated with cancer treatments. Multicenter studies, systematic reviews, and meta-analyses have described high rates of malnutrition in this population, with a particularly high prevalence in pancreatic, gastric, and esophageal cancer. Malnutrition is consistently associated with an increase in postoperative complications, poorer tolerance to cancer treatments, and a significant reduction in overall survival (4–6).

To standardize the diagnosis of malnutrition, the Global Leadership Initiative on Malnutrition (GLIM) proposed a diagnostic framework combining phenotypic and etiologic criteria, with muscle mass loss being a central phenotypic component (7). In digestive oncology, GLIM-defined malnutrition has been linked to increased postoperative morbidity, higher mortality, and poorer clinical outcomes. Nonetheless, the application of GLIM criteria in patients with digestive tumors shows substantial methodological heterogeneity—particularly regarding the assessment technique and the cut-off values used to define low muscle mass—thereby limiting comparability across studies (7–11).

In parallel, sarcopenia has emerged as a key determinant of nutritional risk in digestive oncology. Evidence demonstrates that low skeletal muscle mass is independently associated with higher mortality, impaired survival, and increased postoperative complications in patients with gastrointestinal cancers. Importantly, these alterations may occur even in individuals with normal or elevated body mass index (BMI), underscoring the limitations of traditional anthropometric measures for adequate nutritional risk stratification (12–15).

At the same time, nutritional assessment in oncology has shifted toward a morphofunctional perspective that integrates measures of muscle quantity, quality, and function. Noninvasive and clinically accessible tools—such as bioelectrical impedance analysis (BIA), particularly phase angle; ultrasound of the rectus femoris and abdominal musculature; and assessments of strength and physical function—have shown significant prognostic value in cancer populations, including those with digestive neoplasms (16–19). However, relevant methodological limitations remain, notably the variability in reference standards and cut-off points, and the scarcity of sex-stratified analyses (13, 15).

Given these considerations, there is a clear need to investigate the role of integrated morphofunctional assessment within the GLIM diagnostic framework to identify parameters with independent prognostic value for mortality in patients with digestive cancer.

## 2 Materials and methods

### 2.1 Study design

A retrospective longitudinal observational study was conducted in patients with digestive cancers treated at the Endocrinology and Nutrition outpatient clinics of Puerta del Mar University Hospital (Cádiz, Spain) between May 2021 and July 2025. A total of 131 patients aged ≥18 years with a histologically confirmed diagnosis of digestive cancer (bile ducts, pancreas, esophagus, stomach, colon, sigmoid colon, and rectum) at any stage who were referred for nutritional assessment were included. Patients with incomplete data for key body composition variables or without available follow-up information for survival analysis were excluded.

Cancer diagnosis was verified through medical records and pathological reports, and biopsies were classified by pathologists according to the World Health Organization Classification of Tumors of the Digestive System (2019) (20). Anthropometric and morphofunctional assessments were performed in a standardized manner by trained healthcare personnel during nutritional consultations. Overall survival was determined through clinical follow-up until death or the end of the study period. Patients who declined BIA-based nutritional assessment for reasons including extensive skin lesions, fluid extravasation, local hematomas, amputation, or an estimated life expectancy of <3 months were excluded.

### 2.2 Demographic, clinical, and biochemical variables

Clinical variables included sex (male/female), age (years), BMI (kg/m^2^), tumor location, and clinical stage. Functional status was assessed using the Eastern Cooperative Oncology Group (ECOG) scale (0 = asymptomatic/fully active; 1 = symptomatic but ambulatory; 2 = <50% of the day bedridden; 3 = > 50% bedridden; 4 = completely bedridden; 5 = deceased).

Biochemical variables included serum albumin (g/dL), total protein (g/dL), total cholesterol (mg/dL), glucose (mg/dL), urea (mg/dL), C-reactive protein (CRP, mg/dL), TSH (thyroid-stimulating hormone; μIU/mL), prealbumin (mg/dL), creatinine (mg/dL), and HbA1c (%). Albumin, proteins, cholesterol, glucose, urea, and CRP were quantified using an Alinity c-series biochemistry autoanalyzer (Abbott) via turbidimetric or colorimetric methods. Prealbumin was determined using the bromocresol green method, and creatinine using the kinetic alkaline picrate assay. HbA1c was measured with a Cobas Integra 700 analyzer (Roche Diagnostics) using an immunoturbidimetric method on hemolyzed whole blood, standardized to the NGSP method used in the DCCT and UKPDS trials.

### 2.3 Morphofunctional evaluation

Body composition was evaluated using bioelectrical impedance analysis (BIA) and nutritional ultrasound. Muscle strength was assessed via handgrip dynamometry, and physical performance through the Timed Up and Go (TUG) test.

BIA was performed using a 50 kHz phase-sensitive impedance analyzer (BIA 101 Whole Body Bioimpedance Vector Analyzer, AKERN, Florence, Italy) with tetrapolar electrodes delivering an 800 μA current. Measurements were obtained from the right hand and foot with patients in the supine position after 5 min of rest. The following parameters were recorded: phase angle (PhA, °), standardized phase angle (SPA), body cell mass (BCM, kg), body cell mass index (BCMI, kg/m^2^), fat mass (FM, kg), fat mass index (FMI, kg/m^2^), fat-free mass (FFM, kg), fat-free mass index (FFMI, kg/m^2^), appendicular skeletal muscle mass (ASMM, kg), appendicular skeletal muscle mass index (ASMI, kg/m^2^), total body water (TBW, kg), extracellular water (ECW, kg), intracellular water (ICW, kg), hydration status (%), reactance (Xc, *Ω*/m), and resistance (Rz, Ω/m). Height was measured using a Seca stadiometer (Hamburg, Germany). BIA-derived parameters were standardized by sex and age using reference values from healthy Italian adults (21). PhA was calculated as (Xc/Rz) × (180°/*π*). SPA was derived by subtracting the age- and sex-specific reference PA from the observed PA and dividing the result by the corresponding standard deviation (22). Device accuracy was checked daily using the calibration circuit supplied by the manufacturer, consistently yielding values close to the 385-ohm reference. *In vivo* reproducibility demonstrated coefficients of variation (CV) of 1–2% for Rz and Xc.

A muscle ultrasound of the quadriceps femoris (QRF) was performed using a 10–12 MHz probe and a multi-frequency linear array (Mindray Z60, Madrid, Spain) in all subjects in the supine position. The evaluation was performed without compression in the lower third, between the upper l pole of the patella and the anterior superior iliac spine, measuring the thickness, circumference, and cross-sectional area of the anteroposterior muscle (23). The ultrasound was performed by clinical staff previously trained in this technique. The probe was aligned perpendicular to the longitudinal and transverse axes of the QRF to measure the cross-sectional area of the rectus femoris (RF-CSA), the circumference of the rectus femoris (RF-CIR), the RF axes (X and Y axes), and the subcutaneous fat of the leg (L-SAT). Three measurements were taken for each parameter and the mean was calculated. To assess abdominal adipose tissue, the midpoint between the xiphoid process and the navel was measured, where total subcutaneous abdominal fat (T-SAT), superficial subcutaneous abdominal fat (S-SAT), and preperitoneal or visceral fat (VAT) were measured in centimeters.

To measure handgrip strength (HGS), a Jamar hand dynamometer (Asimow Engineering Co., Los Angeles, CA, USA) was used. Handgrip strength was measured in a seated position with the elbow flexed at 90° in the dominant hand. Patients were instructed to perform three maximum isometric contractions with brief pauses between measurements, and the maximum and average values were recorded. To classify normality, the cut-off points proposed by the *European Working Group on Sarcopenia in Older People 2* (EWGSOP2) criteria were used: men > 27 kg and women > 16 kg.

Functional capacity was assessed using the Timed Up and Go test, which measures the time in seconds it takes to get up from a chair, walk 3 m, turn around, walk another 3 m, and sit back down.

Finally, sarcopenia status was defined using the *EWGSOP2* criteria, considering muscle strength, muscle mass, and physical performance. Muscle strength was assessed using handgrip dynamometry and muscle mass using bioimpedance analysis (appendicular muscle mass normalized for height). Confirmed sarcopenia was defined by the coexistence of low muscle strength and low muscle mass (24).

### 2.4 Nutritional diagnosis

To establish the diagnosis of malnutrition according to the GLIM criteria (7), a combination of phenotypic and etiological criteria was required. The phenotypic criteria used for the diagnosis of moderate cases were: weight loss between 5 and 10% in the last 6 months, BMI < 20 kg/m^2^ in people under 70 years of age, or BMI < 22 kg/m^2^ in patients aged 70 years or older, or FFMI < 17 kg/m^2^ in men or < 15 kg/m^2^ in women. Severe cases were defined as weight loss >10%, BMI < 18.5 kg/m^2^ in individuals <70 years, or BMI < 20 kg/m^2^ in those ≥70 years.

Etiologic criteria included reduced food intake/assimilation and inflammation/disease burden. Reduced intake was defined as <75% of estimated requirements based on a 24-h dietary recall. Gastrointestinal symptoms (dysphagia, nausea, vomiting, diarrhea, constipation, abdominal pain) were assessed as indicators of impaired intake or assimilation. All patients with active tumors requiring chemotherapy or radiotherapy were considered to meet the inflammation criterion (25).

### 2.5 Statistical analysis

Continuous variables were expressed as mean ± standard deviation (SD), and categorical variables as absolute frequencies and percentages.

Two-group comparisons were performed using Student’s t-test or the Mann–Whitney U test depending on data distribution. Comparisons among three groups used one-way ANOVA with *post hoc* Games–Howell tests when appropriate. Categorical variables were compared using the chi-square test. A multivariable logistic regression analysis was performed to evaluate the association between phase angle and nutritional status defined by GLIM criteria (three categories), adjusting for age and sex. Odds ratios (OR) were estimated with their corresponding 95% confidence intervals (CI).

Overall survival was defined as the interval between study inclusion and death from any cause or last follow-up. Survival curves were generated via the Kaplan–Meier method and compared using log-rank tests. The optimal PhA cutoff for mortality prediction was identified using maximally selected rank statistics.

Cox proportional hazards models were constructed to assess associations between nutritional status, PhA, and mortality. The final multivariable model included dichotomized GLIM status (well-nourished vs. malnourished), PhA as a continuous variable, age, and sex. Hazard ratios (HRs) with 95% CIs were reported.

Statistical analysis was performed using Jamovi software (version 2.6.45.0).

## 3 Results

### 3.1 Baseline characteristics of the study population

A total of 131 patients with digestive tumors were included, of whom 73 (55.7%) were men and 58 (44.3%) were women. The mean age was 66 ± 10.4 years and the mean body mass index (BMI) was 23.9 ± 4.37 kg/m^2^. The most common tumor locations were the pancreas (27.3%) and stomach (25%), followed by the esophagus (12.9%) and colon (11.4%). The most prevalent stages were stage IV (25%) and stage III (21.2%). ECOG 0 was the most frequent (46.2%), followed by ECOG 1 (41.5%). During follow-up, overall mortality was 36.7%. There were no significant differences according to sex in terms of location, tumor stage, ECOG, or survival. The complete data are presented in Table 1.

**Table 1:** Baseline characteristics of the study population according to sex.

Variable	All *N =* 131	Males *N =* 73	Females *N =* 58	*p*-value
Demographic variables				
Age (years)	66 (10.4)	65.8 (10.32)	66.3 (10.66)	0.774
BMI (kg/m^2^)	23.9 (4.37)	23.8 (3.88)	23.7 (4.29)	0.934
Cancer location				
Biliary tract	13 (9.8%)	7 (9.6%)	6 (10.2%)	0.352
Pancreas	36 (27.3%)	15 (20.5%)	21 (35.6%)	
Stomach	33 (25%)	18 (24.7%)	15 (25.4%)	
Esophagus	17 (12.9%)	13 (17.8%)	4 (6.8%)	
Colon	15 (11.4%)	8 (11%)	7 (11.9%)	
Sigmoid colon	6 (4.5%)	3 (4.1%)	3 (5.1%)	
Rectum	8 (6.1%)	6 (8.2%)	2 (3.4%)	
Esophagogastric junction	4 (3%)	3 (4.1%)	1 (1.7%)	
Cancer stage				
I	8 (6.1%)	4 (5.5%)	4 (6.8%)	0.562
II	31 (23.5%)	19 (26%)	12 (20.3%)	
III	28 (21.2%)	12 (16.4%)	16 (27.1%)	
IV	33 (25%)	18 (24.7%)	15 (25.4%)	
Not specified	32 (24.2%)	20 (27.4%)	12 (20.3%)	
ECOG				
ECOG 0	60 (46.2%)	31 (43.1%)	29 (50%)	
ECOG 1	54 (41.5%)	33 (45.8%)	21 (36.2%)	
ECOG 2	11 (8.5%)	5 (6.9%)	6 (10.3%)	
ECOG 3	5 (3.8%)	3 (4.2%)	2 (3.4%)	
ECOG 4	0 (0%)	0 (0%)	0 (0%)	
Mortality				
No	81 (63.3%)	46 (66.7%)	35 (59.3%)	0.39
Yes	47 (36.7%)	23 (33.3%)	24 (40.7%)	

Data are expressed as mean ± standard deviations or percentage. Groups were divided by sex variable. Totals may vary due to missing follow-up data in some variables.

### 3.2 Sex-related differences in body composition parameters, muscle ultrasound, and functional performance

Significant sex-related differences were observed in most morphofunctional parameters evaluated (Table 2). In the bioelectrical impedance analysis (BIA), men presented higher values of PhA (4.65 vs. 4.37°; *p* = 0.020), BCM (24.05 vs. 18.4 kg; *p <* 0.001), FFM (52.87 vs. 41.63 kg; *p <* 0.001), ASMM (20.81 vs. 14.76 kg; *p <* 0.001), and ASMMI (7.3 vs. 5.99 kg/m^2^; *p <* 0.001). Women exhibited higher FMI values (6.61 vs. 5.04%; *p* = 0.003).

**Table 2:** Sex-related differences in body composition, muscle ultrasound, and functional parameters.

Variable	All *N =* 131	Males *N =* 73	Females *N =* 589	*P*-value
BIA				
RZ	545 (58.89)	511.52 (74.43)	586.95 (74.71)	< 0.001
XC	43 (8.12)	41.66 (7.66)	44.58 (7.6)	0.037
PhAPA (°)	4.52 (0.72)	4.65 (0.63)	4.37 (0.62)	0.02
SPA	−1.57 (1.07)	−2.1 (1.12)	−0.92 (1.11)	< 0.001
BCM (kg)	21.6 (5.07)	24.05 (3.89)	18.4 (3.96)	< 0.001
FM (kg)	15.3 (7.98)	14.36 (7.43)	16.48 (8.52)	0.13
FFM (kg)	47.8 (8.19)	52.87 (6.33)	41.63 (5.53)	< 0.001
ASMM (kg)	18.1 (4.19)	20.81 (3.01)	14.76 (2.79)	< 0.001
FMI (%)	5.73 (3.04)	5.04 (2.47)	6.61 (3.46)	0.003
FFMI (%)	17.8 (1.86)	18.54 (1.75)	16.87 (1.55)	< 0.001
ASMMI (cm^2^/m^2^)	6.72 (1.17)	7.3 (0.84)	5.99 (1.11)	< 0.001
Echography exploration				
RF-CSA (cm^2^)	3.13 (1.05)	3.5 (1.08)	2.64 (0.79)	< 0.001
RF-CIR (cm)	8.68 (1.14)	9.13 (0.98)	8.1 (1.08)	< 0.001
RF-X axis (cm)	3.65 (0.43)	3.81 (0.33)	3.45 (0.45)	< 0.001
RF-Y axis (cm)	0.95 (0.26)	1.02 (0.27)	0.87 (0.21)	0.002
L-SAT (cm)	0.65 (0.43)	0.45 (0.27)	0.92 (0.45)	< 0.001
T-SAT (cm)	1.26 (0.67)	1.14 (0.58)	1.44 (0.74)	0.062
S-SAT (cm)	0.56 (0.29)	0.48 (0.24)	0.67 (0.34)	0.007
VAT (cm)	0.38 (0.25)	0.36 (0.22)	0.42 (0.29)	0.317
Functional measurement				
HGS max (kg)	22.5 (9.06)	26.62 (7.91)	17.01 (7.51)	< 0.001
HGS mean (kg)	20.8 (8.82)	25.04 (7.87)	15.26 (6.71)	< 0.001
TUG (s)	6.45 (1.15)	6.20 (1.04)	6.81 (1.22)	0.055
Sarcopenia				
No	110 (84.6%)	57 (79.2%)	53 (91.4%)	0.055
Yes	20 (15.4%)	15 (20.8%)	5 (8.6%)	

Data are expressed as mean ± standard deviations or percentage. Groups were divided by sex variable. BIA, Bioelectrical impedance analysis; FM, Fat mass; HGS, Hand grip strength; PhA, Phase angle; RF-CIR, Circumference of quadriceps rectus femoris; RF-CSA, Rectus femoris cross-sectional area; SAT, Subcutaneous adipose fat of leg (L), superficial (S), and total (T) abdominal; VAT, Preperitoneal or visceral fat; SMI, Skeletal muscle index; SPA, Standardized phase angle; TUG, Timed Up and Go.

In the ultrasound assessment, men showed higher RF-CSA (3.5 vs. 2.64 cm^2^; *p <* 0.001) and RF-CIR (9.13 vs. 8.1 cm; *p <* 0.001), whereas women had higher L-SAT values (0.92 vs. 0.45 cm; *p <* 0.001).

Regarding functional tests, men demonstrated higher maximal HGS (26.62 vs. 17.01 kg; *p <* 0.001) and mean HGS (25.04 vs. 15.26 kg; *p <* 0.001). No significant differences were observed in TUG performance (*p* = 0.055).

The prevalence of sarcopenia in the total sample was 15.4%, with no significant sex-related differences (p = 0.055). Complete data are presented in Table 2.

In the biochemical analysis, sex-related differences were limited to creatinine levels (0.87 ± 0.43 vs. 0.70 ± 0.29 mg/dL; *p* = 0.016), which were higher in males, and total cholesterol (185.64 ± 65.97 vs. 158.91 ± 40.71 mg/dL; p = 0.01), which was higher in females, with no significant differences observed in the other parameters evaluated. The detailed results stratified by sex are presented in the Supplementary Table S1.

### 3.3 Nutritional classification according to GLIM criteria

The study population was stratified into three groups according to GLIM-defined nutritional status: well-nourished (*N =* 18), moderately malnourished (*N =* 58), and severely malnourished (*N =* 55) (Table 3).

**Table 3:** Baseline characteristics of the study population divided into three categories according to the GLIM variable.

Variable	Well-Nourished *N =* 18	Moderate *N =* 58	Severe *N =* 55	*P-*value
Demographic variables				
Age (years)	63.6 (12)	68.9 (9.84) ^c*^	63.8 (10)	0.043
Sex (male/female)	12/6	28/32	33/22	0.200
BMI (kg/m^2^)	27 (3.24) ^a*, b***^	24.5 (3.57) ^c***^	21.4 (3.24)	< 0.001
BIA				
RZ	400 (66.1) ^b**^	546 (86.2)	560 (61.8)	0.006
XC	41.8 (8.46)	43.6 (8.7)	42.8 (7.27)	0.744
PhA (°)	4.78 (0.72)	4.58 (0.66)	4.39 (0.71)	0.121
SPA	−1.45 (1.24)	−1.39 (1.25)	−1.79 (1.23)	0.243
BCM (kg)	24.9 (5.75) ^b*^	21.4 (4.7)	20.8 (4.91)	0.032
FM (kg)	21.3 (8.17) ^b***^	16.7 (7.48) ^c**^	12.1 (6.93)	< 0.001
FFM (kg)	53.2 (9.03) ^a*, b*^	47 (7.98)	46.8 (7.63)	0.029
ASMM (kg)	20.8 (4.41) ^a*,b*^	17.7 (4.12)	17.6 (3.96)	0.028
FMI (%)	7.73 (2.72) ^b***^	6.45 (3.13) ^c***^	4.38 (2.43)	< 0.001
FFMI (%)	19.3 (1.8) ^b***^	18.1 (1.92) ^c**^	17 (1.42)	< 0.001
ASMMI (cm^2^/m^2^)	7.47 (1.02) ^b**^	6.78 (1.14)	6.42 (1.15)	0.003
Echography exploration				
RF-CSA (cm^2^)	3.86 (1.32) ^a*^	2.93 (0.84)	3.1 (1.05)	0.042
RF-CIR (cm)	8.83 (1.44)	8.56 (1.08)	8.73 (1.12)	0.714
RF-X axis (cm)	3.63 (0.54)	3.64 (0.41)	3.67 (0.40)	0.930
RF-Y axis (cm)	1.21 (0.28) ^a**, b**^	0.92 (0.20)	0.91 (0.25)	0.002
L-SAT (cm)	0.66 (0.35)	0.76 (0.45)	0.58 (0.42)	0.157
T-SAT (cm)	1.56 (0.77)	1.26 (0.66)	1.14 (0.61)	0.271
S-SAT (cm)	0.64 (0.36)	0.56 (0.28)	0.52 (0.29)	0.574
VAT (cm)	0.52 (0.33)	0.37 (0.20)	0.34 (0.24)	0.264
Functional measurement				
HGS max (kg)	24.1 (6.84)	21.1 (10.3)	23.1 (8.79)	0.503
HGS mean (kg)	21.8 (6.3)	19.3 (10.2)	21.7 (8.46)	0.543
TUG (s)	6.71 (1.37)	6.33 (1.14)	6.50 (1.12)	0.760
Sarcopenia				
No	18 (100%)	51 (91.1%) ^c*^	41 (74.5%)	0.008
Yes	0	5 (8.9%)	14 (25.5%)	
Mortality				
No	16 (88.9%) ^a*^	32 (57.1%)	32 (60.4%)	0.046
Yes	2 (11.1%)	24 (42.9%)	21 (39.6%)	

Data are expressed as mean ± standard deviations or percentage. Groups were divided by GLIM nutritional status being well-nourished, moderate, and severe malnutrition, the groups included in the study. Asterisk indicates significant difference between groups, according to the ANOVA test followed by pairwise comparisons adjusting for Games-Howell method (Chi-squared test was used for variables expressed as a percentage) (***p < 0.001, **p < 0.01, *p < 0.05). ^a^*p*-value, Well-nourished vs. moderate; ^b^*p*-value, Well-nourished vs. severe; ^c^*p*-value, moderate vs. severe. Totals may vary due to missing follow-up data in some variables. BIA, Bioelectrical impedance analysis; FM, Fat mass; HGS, Hand grip strength; PhA, Phase angle; RF-CIR, Circumference of quadriceps rectus femoris; RF-CSA, Rectus femoris cross-sectional area; SAT, Subcutaneous adipose fat of leg (L), superficial (S), and total (T) abdominal; VAT, Preperitoneal or visceral fat; SMI, Skeletal muscle index; SPA, Standardized phase angle; TUG, Timed Up and Go.

Significant differences in BMI were observed across all groups (*p <* 0.001), including differences between moderate and severe malnutrition.

Patients with moderate or severe malnutrition exhibited significantly lower values in several BIA-derived parameters, including BCM, FFM, ASMM, and their corresponding indices (*p <* 0.05). FM, FMI, and FFMI also differed significantly between the two malnutrition groups, with lower values in patients with severe malnutrition.

In the ultrasound assessment, RF-CSA and the RF-Y axis differed significantly across nutritional severity categories (*p <* 0.05).

The prevalence of sarcopenia was significantly higher in the severe malnutrition group compared with the moderate group (*p <* 0.05). No significant differences were observed in functional performance tests. Full data are presented in Table 3.

### 3.4 Association between phase angle and nutritional severity according to GLIM criteria

Multivariable logistic regression models were conducted to evaluate the association between PhA and GLIM-defined malnutrition categories using pairwise comparisons (Table 4). All models were adjusted for age and sex.

**Table 4:** Multivariable logistic regression analysis of GLIM-defined nutritional status (pairwise comparisons).

Variable	Well-nourished vs. moderate malnutrition OR (95% CI)	*P*	Well-nourished vs. severe malnutrition OR (95% CI)	*P*	Moderate vs. Severe malnutrition OR (95% CI)	*P*
Age (years)	1.05 (0.99–1.11)	0.124	1.03 (0.97–1.09)	0.300	1.08 (1.03–1.13)	0.002
Phase angle (°)	1.13 (0.47–2.70)	0.789	2.34 (0.93–5.94)	0.072	2.51 (1.26–4.99)	0.009
Female sex	2.85 (0.90–9.07)	0.076	0.85 (0.27–2.68)	0.779	2.29 (1.02–5.19)	0.046

GLIM-defined nutritional status (well-nourished, moderate malnutrition, and severe malnutrition) was analyzed using pairwise multivariable logistic regression models. Odds ratios (OR) with 95% confidence intervals were estimated after adjustment for age and sex. Phase angle was modeled as a continuous variable. Reference category for the dependent variable: severe malnutrition.

When comparing moderate versus severe malnutrition, PhA was independently associated with GLIM-defined nutritional severity (OR = 2.51; 95% CI: 1.26–4.99; *p* = 0.009).

No significant association was found between well-nourished and moderately malnourished patients (*p* = 0.789). A non-significant trend was observed between well-nourished and severely malnourished patients (*p* = 0.072).

Age and female sex were independently associated with greater nutritional severity in the comparison between moderate and severe malnutrition (*p* = 0.002 and *p* = 0.046, respectively).

### 3.5 Overall survival according to GLIM criteria

**Figure 1:**
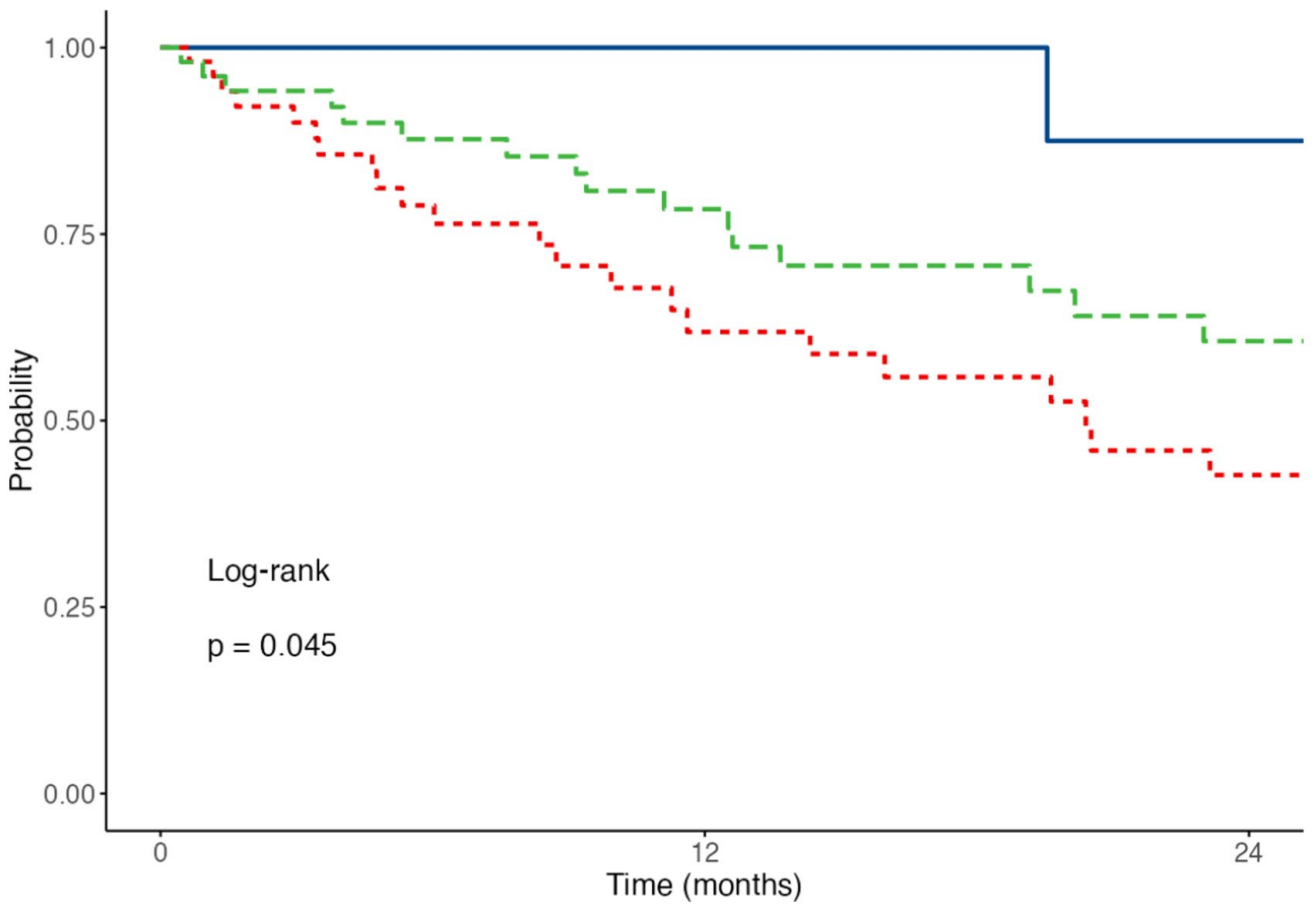
Overall survival according to GLIM nutritional status. Kaplan–Meier survival curves comparing overall survival among GLIM nutritional categories: well-nourished (blue solid line), moderate malnutrition (red dashed line), and severe malnutrition (green dashed line). Differences were assessed using the log-rank test.

Kaplan–Meier curves demonstrated significant differences in overall survival according to GLIM-defined nutritional status (log-rank *p <* 0.05) (Figure 1).

Well-nourished patients showed the greatest overall survival, with a median of 38.5 months, compared with 20.4 months in patients with moderate malnutrition and 26.2 months in those with severe malnutrition.

### 3.6 Morphofunctional parameters and mortality

**Figure 2:**
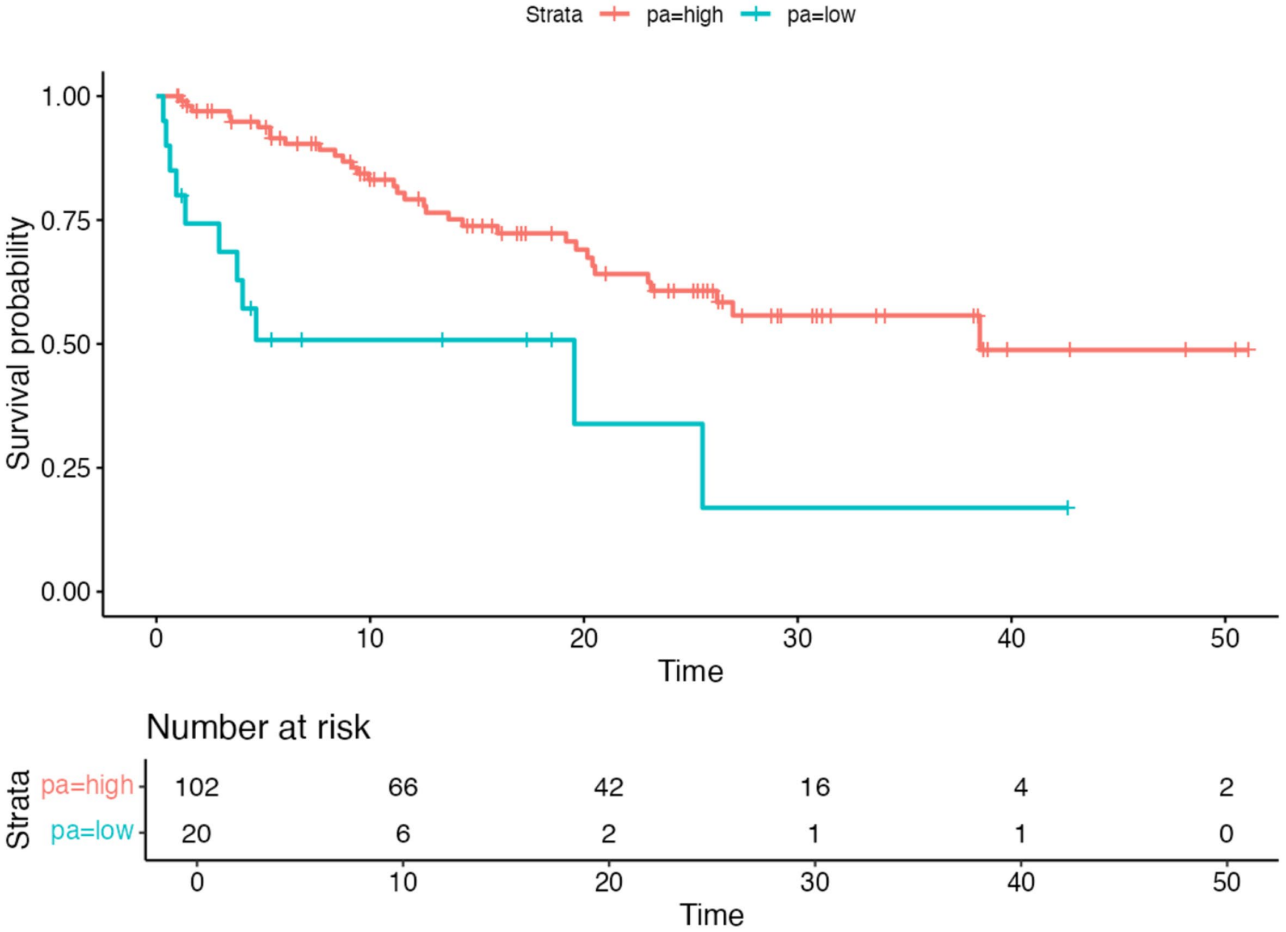
Overall survival according to phase angle cut-off. Kaplan–Meier survival curves according to phase angle cut-off. Differences were assessed using the log-rank test.

To determine an optimal prognostic threshold for phase angle in the overall cohort, the *maximally selected rank statistics* method was applied, yielding a cutoff point of 3.8°. The median survival was 38.5 months in the group with PhA ≥ 3.8° compared to 10.5 months in the group with PhA < 3.8° (Figure 2).

In the Cox regression model, PhA analyzed as a continuous variable was significantly associated with mortality (HR = 0.59; 95% CI: 0.36–0.97; *p* = 0.038).

### 3.7 Multivariate survival analysis

A multivariable Cox regression model was developed to identify independent predictors of all-cause mortality (Table 5). GLIM nutritional status was included as a dichotomous variable (well-nourished vs. malnourished).

**Table 5:** Multivariable cox regression analysis for all-cause mortality.

Variable	Comparison	HR (multivariable)	*P*
GLIM status	Malnourished vs. well-nourished	4.35 (1.04–16.67)	0.044
PhA	Per 1° increase	0.57 (0.34–0.97)	0.039
Age	Per 1-year increase	1.02 (0.99–1.05)	0.214
Sex	Female vs. Male	0.88 (0.49–1.58)	0.672

Hazard ratios (HR) and 95% confidence intervals (CI) were estimated using a multivariable Cox proportional hazards model adjusted for age, sex, and phase angle.

Malnutrition remained independently associated with increased mortality after adjusting for age, sex, and PhA (HR = 4.35; 95% CI: 1.04–16.67; *p* = 0.044).

PhA also showed an independent association with survival (HR = 0.57; 95% CI: 0.34–0.97; *p* = 0.039). Neither age nor sex were significantly associated with mortality in the final model.

## 4 Discussion

In this study, GLIM-defined malnutrition showed a high prevalence among patients with digestive neoplasms and was independently associated with an increased risk of overall mortality. Additionally, PhA measured by BIA emerged as an independent prognostic marker, even after adjusting for nutritional status, age, and sex.

Our results extend previous evidence on the prognostic impact of disease-related malnutrition in digestive oncology. Prior studies in patients with gastrointestinal cancers have demonstrated that malnutrition is associated with poorer treatment tolerance, higher complication rates, and lower survival (3–6, 26–28). Consistent with this evidence, our multivariable model showed that malnourished patients had a significantly higher risk of mortality than well-nourished individuals, reinforcing the clinical value of GLIM criteria in this population. At the same time, the broad confidence interval observed for this association suggests limited precision and indicates that this finding should be interpreted with caution rather than as a definitive estimate of effect size.

A key strength of the present study is the integration of a comprehensive morphofunctional assessment. Malnourished patients showed worse body composition parameters and a higher prevalence of sarcopenia, highlighting the central role of muscle impairment in cancer prognosis. Substantial evidence in digestive oncology has shown that low skeletal muscle mass is consistently associated with higher mortality and poorer outcomes (12–16).

In this context, accessible and reproducible tools such as BIA and muscle ultrasound may facilitate the practical implementation of GLIM criteria in routine clinical settings.

A relevant finding is that PhA showed an independent association with survival in both the cutoff point analysis and the continuous Cox model. In our cohort, lower PhA values were associated with lower overall survival, and this relationship remained after adjustment for relevant clinical variables and GLIM-defined nutritional status. These findings support the role of PhA as an additional prognostic marker in patients with digestive cancer. Previous studies in oncology populations, including digestive tumors, have consistently identified PhA as an independent predictor of mortality and worse clinical outcome (16, 19, 29, 30) in agreement with our results.

In the GLIM category analysis, although clear morphofunctional differences were observed between moderate and severe malnutrition, the survival gradation did not show a strictly linear progression between the two groups. This finding should be interpreted cautiously. It may be related to sample size limitations and to the clinical heterogeneity of the cohort. Similar findings have been described in previous studies applying GLIM criteria in cancer populations, where stratification by severity grades does not always translate into proportional differences in mortality (9, 10, 27).

From a clinical perspective, our findings support the systematic incorporation of structured nutritional assessment in patients with digestive cancer, as recommended by international guidelines (8). In this context, GLIM-defined malnutrition showed clear prognostic value, while phase angle (PhA) provided additional and independent information for risk stratification. The integration of GLIM criteria with morphofunctional parameters such as PhA may therefore improve the identification of clinically vulnerable patients and enhance prognostic stratification in routine clinical practice.

Among the main strengths of the study is the protocolized application of a comprehensive morphofunctional assessment, carried out in a real healthcare setting by trained personnel. This approach reflects routine clinical practice and supports the external applicability of our findings to other centers with comparable clinical settings and resources. However, several limitations should be considered. First, this was a retrospective study, which limits causal inference. Second, the cohort was clinically heterogeneous in terms of tumor location and stage, and these factors may have influenced both the nutritional comparisons and the survival analyses. Third, although the multivariable Cox model included age, sex, GLIM-defined malnutrition, and PhA, other relevant oncologic prognostic variables such as tumor stage, ECOG performance status, and tumor location were not included in the final model; therefore, residual confounding cannot be excluded. In addition, the inclusion of patients referred to the nutrition unit may have introduced selection bias and limited the representativeness of the sample. Finally, the 3.8° PhA cutoff was derived within the same cohort using maximally selected rank statistics and should therefore be regarded as an exploratory internally derived threshold requiring external validation.

## 5 Conclusion

Malnutrition defined by GLIM criteria is common in patients with digestive cancer and is independently associated with poorer overall survival. Phase angle, as a morphofunctional parameter derived from BIA, provides additional prognostic value and improves the identification of patients at higher risk. These findings support the need to systematically integrate morphofunctional nutritional assessment into the care of patients with digestive neoplasms, with the aim of improving risk stratification and optimizing nutritional intervention strategies.

## Data Availability

The original contributions presented in the study are included in the article/Supplementary material, further inquiries can be directed to the corresponding authors.

## Glossary

GLIM: Global Leadership Initiative on Malnutrition
BIA: bioelectrical impedance analysis
PhA: phase angle
SPA: standardized phase angle
BCM: body cell mass
BCMI: body cell mass index
FFM: fat-free mass
FFMI: fat-free mass index
FM: fat mass
FMI: fat mass index
ASMM: appendicular skeletal muscle mass
SMI: Skeletal muscle index
TBW: total body water
ECW: extracellular water
ICW: intracellular water
RZ: resistance
XC: reactance
BMI: body mass index
ECOG: Eastern Cooperative Oncology Group
CRP: C-reactive protein
TUG: Timed Up and Go
HGS: handgrip strength
QRF: quadriceps femoris
RF-CSA: area of the rectus femoris
RF-CIR: circumference of the rectus femoris
RF-X axis: X axis of the rectus femoris
RF-Y axis: Y axis of the rectus femoris
L-SAT: subcutaneous adipose fat of the leg
T-SAT: total subcutaneous abdominal fat
S-SAT: superficial subcutaneous abdominal fat
VAT: preperitoneal or visceral fat
EWGSOP2: European Working Group on Sarcopenia in Older People
OR: Odds ratios
HR: Hazard ratios
SD: standard deviation
CI: confidence intervals
