# Supplementary material for: Prognostic impact of glim-defined malnutrition and phase angle in patients with digestive tumors
Source: Front Nutr. 2026 May 13;13:1836804. doi: 10.3389/fnut.2026.1836804 (PMC13212207; doi:10.3389/fnut.2026.1836804)
Supplement: Supplementary file 1 [file Table_1.docx]

Supplementary Material

**Table S1.** Sex-related differences in biochemical variables.

|  | All  N=131 | Males  N=73 | Females  N=59 | P value |
| --- | --- | --- | --- | --- |
| *Biochemical variables* |  |  |  |  |
| Glucose (mg/dL)  Creatinine (mg/dL)  Urea (mg/dL)  Proteins (g/dL)  Albumin (g/dL)  Pre-albumin (mg/dL)  hs-CRP (mg/dL)  HbA1c (%)  Total cholesterol (mg/dL)  TSH (μUI/mL) | 109 (34.6)  0.80 (0.39)  35.9 (18.2)  6.72 (0.81)  3.94 (0.58)  26.7 (38.7)  29.6 (48.6)  6.23 (1.45)  171 (55)  2.04 (1.84) | 107.29 (33.19)  0.87 (0.43)  37.01 (16.12)  6.66 (0.79)  3.93 (0.59)  21.73 (10.21)  29.82 (49.55)  6.27 (1.63)  158.91 (40.71)  1.75 (1.21) | 110.6 (36.59)  0.70 (0.29)  34.49 (20.72)  6.78 (0.82)  3.94 (0.57)  32.08 (55.54)  29.21 (48.03)  6.16 (1.23)  185.64 (65.97)  2.31 (2.29) | 0.597  0.016  0.457  0.412  0.976  0.515  0.955  0.766  0.010  0.379 |

*Data are expressed as mean ± standard deviations or percentage. Groups were divided by sex variable.*
